# Bio-inspired Composite Microfibers for Strong and Reversible Adhesion on Smooth Surfaces

**DOI:** 10.1093/icb/icz009

**Published:** 2019-04-27

**Authors:** D -M Drotlef, C B Dayan, M Sitti

**Affiliations:** Physical Intelligence Department, Max Planck Institute for Intelligent Systems, Stuttgart 70569, Germany

## Abstract

A novel approach for high-performance gecko-inspired adhesives for strong and reversible adhesion to smooth surfaces is proposed. The composite patterns comprising elastomeric mushroom-shaped microfibers decorated with an extremely soft and thin terminal layer of pressure sensitive adhesive. Through the optimal tip shape and improved load sharing, the adhesion performance was greatly enhanced. A high adhesion strength of 300 kPa together with superior durability on smooth surfaces are achieved, outperforming monolithic fibers by 35 times. Our concept of composite microfibrillar adhesives provides significant benefits for real world applications including wearable medical devices, transfer printing systems, and robotic manipulation.

## Introduction

Nature offers inspiring strategies for strong and reversible adhesion to complex environments. For instance, geckos can reversibly adhere to various surfaces with their adhesive pads covered by dense array of fine curved setae decorated with spatula (Autumn et al. 2000, 2002; Autumn 2006). For the past decades, gecko-inspired adhesives have been extensively studied for adhesion to both smooth and rough surfaces. Their adhesion enhancement by contact splitting (Arzt et al. 2003), contact geometry (Kim and Sitti 2006; del Campo et al. 2007), or the mechanism of equal load sharing (Carbone and Pierro 2012; Aksak et al. 2014) are well understood. Consequently, artificial mimics demonstrate strong and reversible adhesion and even surpass the adhesion performance of the gecko on smooth surfaces (Ge et al. 2007; Murphy et al. 2009; Drotlef et al. 2014).

Recently, the composition of animals’ setae has revealed their mechanically graded structure (Peisker et al. 2013). Additionally, it has been reported that softening of the setae upon variations in environmental conditions (e.g., humidity) can improve the adhesion performance (Puthoff et al. 2010; Prowse et al. 2011). Inspired from these recent findings, composite microfibers with a continuous soft layer on the top of their tips (film-terminated fibers) (Shahsavan and Zhao 2014), hard fiber cores and soft shell (Minsky and Turner 2015), and microfibers with hard fibers and soft tips (Fischer et al. 2017; Gorumlu and Aksak 2017) have been demonstrated. Recently, we have shown that direct crosslinking of composite microfibers decorated with viscous tips on various surfaces can greatly boost the adhesion (Drotlef et al. 2017). However, the reversible adhesion performance of these composite architectures is still challenging.

Here, we propose bio-inspired composite microfibers for strong and reversible adhesion to smooth surfaces. The proposed microfibrillar patterns are composed of polydimethylsiloxane (PDMS) fibers decorated with vinylsiloxane (VS) mushroom-shaped tips, which are additionally coated with an extremely soft and thin terminal layer of silicone-based pressure sensitive adhesive (S-PSA). We demonstrate that mushroom tips coated with thin S-PSA terminal layers can greatly enhance the adhesion through their optimal shape and enhanced load sharing. A high adhesive strength of 300 kPa together with superior reversibility on planar surfaces are achieved after tailoring the tip geometry, tip composition and terminal layer thickness, and tip edge sharpness.

## Results and discussion

**Fig. 1:**
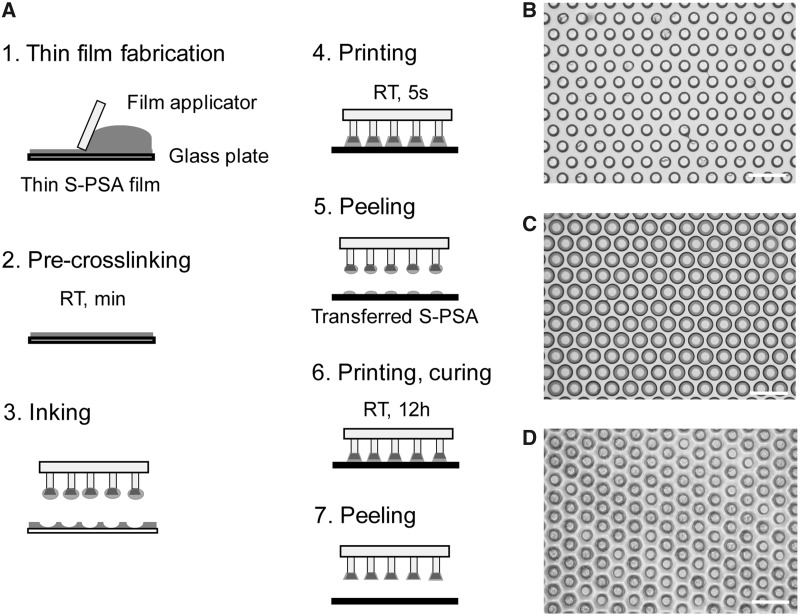
Fabrication process of the microfibrillar mushroom patterns (**A**); fabrication and precuring of the thin S-PSA film (Steps 1–2), inking and printing of microfibrillar patterns onto a silicon wafer (Steps 3–4), second printing process (double printed) on a pristine wafer, curing, and peeling (Steps 6–7). Microscopy images of the fabricated patterns with S-PSA mushroom tip (**B**), double printed composite mushroom fibers with optimally shaped tips (**C**), and film-terminated patterns (**D**). Scale bars: 200 μm.

The fabrication process of composite micropatterns is illustrated in Fig. 1 (see the “Materials and Methods” section for details). Composite micropatterns with various tip geometries and tip compositions were obtained by employing soft molding techniques (del Campo et al. 2007; Drotlef et al. 2013). Briefly, the S-PSA precursor solution was first cast onto a glass plate and a thin and homogeneous film was obtained by a film applicator (Step 1). After partial crosslinking of the S-PSA layer (Step 2), the PDMS microfibers with VS mushroom-shaped tips were manually inked onto the thin layer, leading to the selective transfer of the viscous S-PSA onto microfiber tips (Step 3). The microfibers coated with S-PSA were then printed onto a salinized silicon wafer (Step 4), peeled-off (Step 5), printed a second time on a pristine wafer, and cured at room temperature for 12 h (Step 6). Finally, the patterns were carefully peeled-off from wafer and homogeneous composite micropatterns with a diameter of 67 µm, spacing of 33 µm, and height of 95 µm were fabricated (Step 7).

We selected S-PSA as our tip coating material due to its several key characteristics. First, S-PSA is much softer than PDMS and has a Young’s modulus of ca. 75 kPa, enabling superior conformation to various surfaces or skin. The Young’s modulus was determined from load–displacement curves (Greiner et al. 2007). Second, its suitable viscosity enables efficient inking and printing process. Third, it belongs to the family of silicone polymers and allows covalent bonding with the PDMS. Last, S-PSA is developed and approved for biomedical applications and may be suited for skin related applications.

**Fig. 2:**
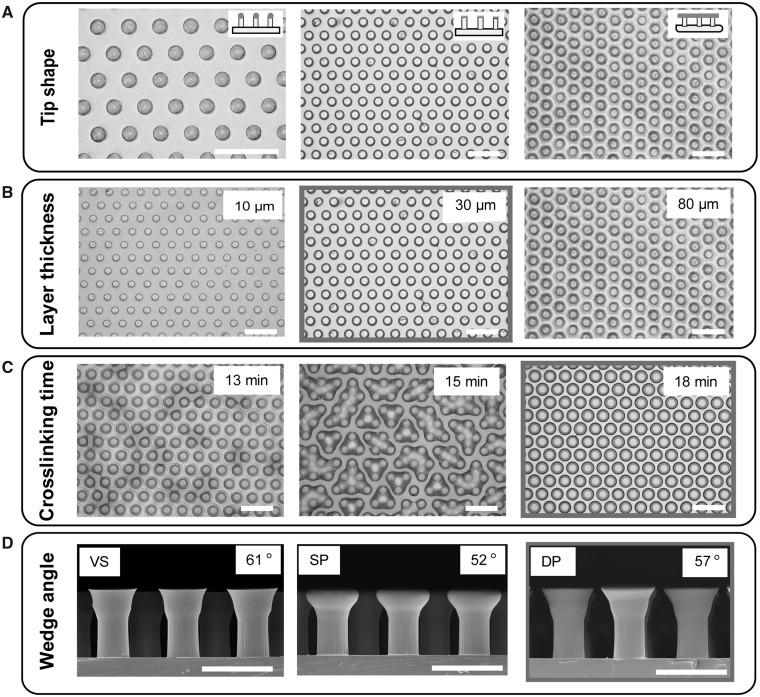
Optimization of the process parameters, including microfibrillar tip shape, layer thickness, pre-crosslinking duration, and wedge angle. Optical microscopy images of patterns with different tip geometry (**A**). Microscopy images of microfibrillar patterns with small and medium S-PSA mushroom tips and micropatterns fully embedded in S-PSA, after inked onto S-PSA films with different thickness (**B**). Microscopy images of composite mushroom microfibers inked into S-PSA films after different pre-crosslinking durations (**C**). Cross-sectional SEM images of mushroom patterns before (VS), after the single (SP) and the DP process, showing fiber tip geometries with varying wedge angles and wedge sharpness **(D)**. Optimal process parameters are indicated with gray borders. Scale bars: (A–C) 200 µm and (D) 100 µm.

**Fig. 3:**
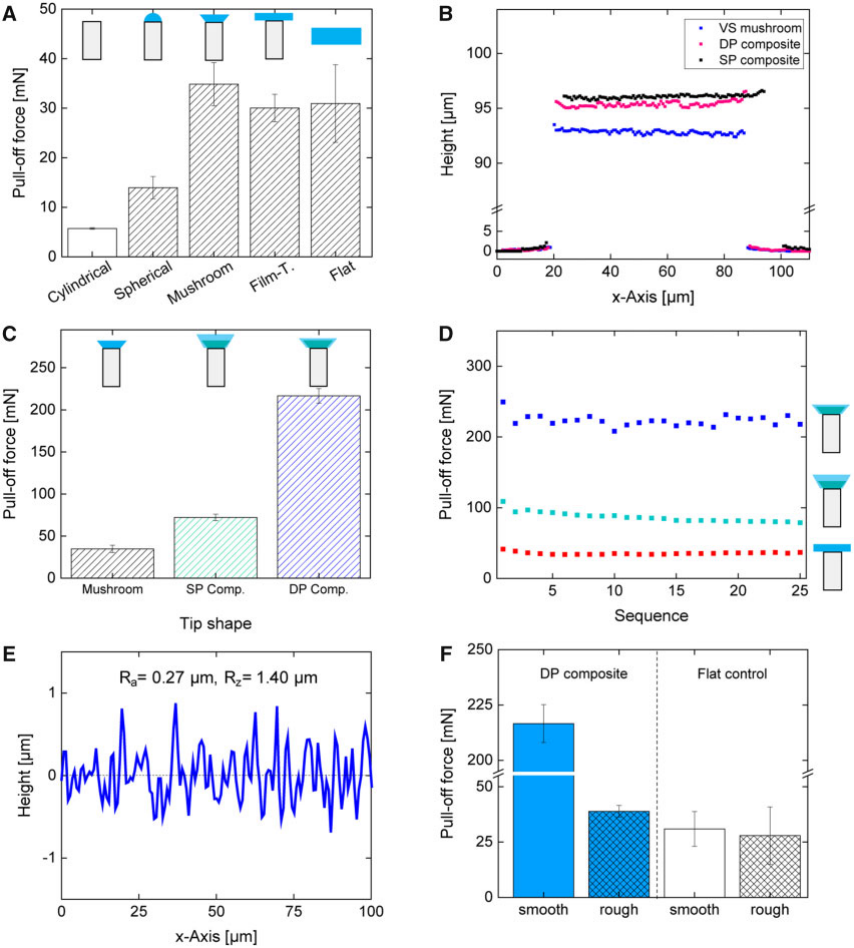
Adhesion measurements of microfibers with various tip geometries and the mushroom composite microfibrillar adhesives with height profiles measured with a 3D laser-scanning microscope. Pull-off force of microfibers with different tip geometries and a flat S-PSA control, measured on a smooth substrate (**A**). 3D laser-scanning microscope height profiles of mushroom patterns before (VS mushroom), after the SP or the DP process (**B**). Pull-off force of microfibers with S-PSA mushroom tips and SP- and double-printed composite mushroom patterns (**C**). Pull-off force of SP and double printed composite mushroom fibers in a durability test over 25 adhesion cycles, compared with film-terminated microfibers (**D**). Surface profile of the rough probe employed for adhesion characterizations with an arithmetical mean deviation (*R*_a_) of 0, 27 µm and mean peak-to-valley roughness (*R*_z_) of 1.4 µm, obtained by 3D laser scanning microscopy (**E**). Pull-off force of double printed composite fibers measured on smooth and rough surfaces, compared with a flat S-PSA control (**F**).

To optimize the shape and layer thickness of multilayer composite microfibers, we prepared fibers with different tip geometries by varying the inking and printing process, the layer thickness of the S-PSA, and the pre-crosslinking duration. Consequently, we evaluated the surface topography of all adhesive patterns, the quality of the coated tips (Fig. 2), and finally their adhesion performance (Fig. 3).

Inspired by fine hairs of various animals comprising different tips geometries ( e.g., flat, hemispherical, conical, toroidal, filament-, band-, and suction cup-like shapes; Spolenak et al. 2005), together with modification of previously reported soft molding techniques (del Campo et al. 2007), microfibers with hemispherical tips, S-PSA mushroom fibers, film-terminated fibers (Fig. 2A), composite mushroom fibers with different S-PSA terminal layer thickness, tip wedge angles, and tip sharp edges were obtained.

As shown in Fig. 2B, S-PSA films with layer thickness ranging from 25 to 30 µm resulted in homogeneous and medium sized mushroom-shaped tips. For thinner layers, however, the transferred S-PSA to the microfiber tips was small and formed small mushroom tips. On the other hand, microfibers were fully immersed in S-PSA when the layer thickness approached or surpassed the fiber height.

Further, we found that 7–9 min was the ideal pre-crosslinking time range for mushroom patterns with medium sized and homogeneous tips. For shorter pre-crosslinking time, the initial viscosity was low and the amount of the transferred S-PSA to the tip of microfibers was large, leading to film-terminated, inhomogeneous, and connected microfiber tips. Moreover, the viscosity was high for long pre-crosslinking duration and thus a small amount of S-PSA was transferred to tips.

Next, homogeneous and large composite mushroom-shaped tips with 2 µm thin S-PSA layers were formed when mushroom patterns with VS tips were inked after 14–15 min pre-crosslinking time (see Fig. 2C). In contrast, connected and island-like microfiber tips were formed for shorter pre-crosslinking durations (see the “Materials and Methods” section for details).

Finally, composite mushroom patterns with sharp tip edges and a tip wedge angle of 57° were obtained by the double printing (DP) process, while composite mushroom patterns with rounded tip edges and a tip wedge angle of 52° were obtained by the single printing process (see Fig. 2D). Notably, VS mushroom patterns have sharp tip edges and a tip wedge angle of 61° prior the inking and printing process.

To investigate the effect of tip geometry on adhesion, the force–displacement curves were measured by a customized adhesion setup (see the “Materials and Methods” section for details). Figure 3A illustrates the adhesion of microfibrillar adhesives with different tip geometries. The maximum adhesion force of 35 mN was achieved by homogeneous and large mushroom-shaped S-PSA tips, while smaller and deformed tips exhibit a lower adhesion performance. Furthermore, patterns with planar PDMS fibers, spherical, film-terminated tips, and a flat S-PSA control showed 6, 14, 30, and 31 mN, respectively. In comparison to patterns with cylindrical uncoated tips, mushroom patterns show a six-fold increase of the adhesive strength.

Similar observations were reported in previous studies, demonstrating the superior adhesion of mushroom fiber over other tip geometries (del Campo et al. 2007; Drotlef et al. 2013). However, our results prove that the same behavior applies for composite mushroom fibers. The superior adhesion performance of mushroom-shaped fibers originates from the more uniform stress distribution at the fiber tip interfaces (Kim and Sitti 2006; Carbone and Pierro 2012). The higher adhesion performance of larger mushroom tips is attributed to their optimized geometry and subsequent improved load sharing (Carbone and Pierro 2012; Marvi et al. 2015).

At this point, it is of interest to compare our S-PSA mushroom micropatterns to related composite adhesives in literature. In a macroscopic approach, a stiff fiber core with 1.25 mm diameter was covered by a compliant PDMS shell with varying tip layer thickness between 100 and 1500 µm (Minsky and Turner 2015). By introducing of a stiff core, an adhesion enhancement by the factor of 3 compared with monolithic soft sample was observed. In another work, a 2 mm diameter stiff fiber stalk with a softer tip layer varying between 20 and 500 µm thickness was studied (Fischer et al. 2017). It was found that composite pillars improved the adhesion to the smooth substrates by a factor 2–3 compared with conventional pillar structures made from monolithic material.

In our experiments, we found a similar improvement by a factor of 2 for fibers with a hemispherical S-PSA tip compared with monolithic PDMS fibers. However, when we tested mushroom fibers with S-PSA tips, a six-fold enhancement was observed largely due to optimal tip shape and improved load sharing.

Although our S-PSA mushroom fibers demonstrated enhanced performance, the size and shape of the tip could not be further altered without causing folding of the thin mushroom rim or fiber coalescence. To overcome these limitations, instead of solely cylindrical fibers, mushroom fibers with VS tips were employed in the inking and printing process. Thereby, large and homogeneous composite mushroom fibers with a thin S-PSA terminal layer were obtained. The stiffer mushroom tip supports soft S-PSA layer, allowing homogeneous and robust tips, suppressing the folding of the delicate rim. These multilayered composite fibers exhibit an improve adhesion force of 70 mN which is 2 and 12 times higher than S-PSA mushroom and monolithic PDMS patterns, respectively (Fig. 3C).

Although these patterns demonstrate enhanced adhesion, the overall quality and homogeneity of the patterns were further improved by employing a double-printing (DP) process. Here, the S-PSA-coated VS mushroom patterns were peeled-off directly after the first printing process, before the S-PSA polymerized. Thereby S-PSA precursor solution was transferred from the fiber tip to the substrate, resulting in a reduced S-PSA layer thickness on the fiber after printing on a pristine substrate.

Laser scanning microscope characterizations revealed that the thickness of the S-PSA layer on the VS mushrooms patterns was reduced from 2 to 1 µm through the DP process (Fig. 3B). Further, cross-sectional SEM characterization of mushroom patterns showed that mushroom fiber tips of the DP composite patterns have sharp edges and a wedge angle of 57°, while SP composite patterns have rounded tip edges and a tip wedge angle of 52°. This led to an extremely high adhesion force of 200 mN due to the optimal and very homogeneous shape of the composite mushroom patterns, leading to 7 and 35 times further adhesion improvement compared with S-PSA mushroom and monolithic PDMS fibers, respectively (Fig. 3C). Notably, the combination of a thin S-PSA terminal layer together with sharp tip edges of the composite fibers is more beneficial than a thicker S-PSA terminal layer with rounded tip edges.

Our results show a three-fold adhesion increase for the DP composite fibers with 1 µm layer thickness which is in agreement with experimental and theoretical findings, where the effect of layer thickness on the adhesion performance was investigated. For example, it was shown that the pull-off stress increased with decreasing the S-PSA film thickness; particularly, a two-fold increase of the pull-off stress was obtained for 50 µm thin films compared with 230 µm films (Fischer et al. 2017).

In other works, it was theoretically predicted that for very thin films, the critical pull-off stress scales with (*E*/*t*)^1/2^, where *E* is the Young’s modulus and *t* the thickness of the thin film. Therefore, the pull-off force is maximized for a thin layer with a low modulus (Chung and Chaudhury 2005). Similar results have been reported by employing finite element modeling. The pull off force increased when the thickness of a soft layer on a stiffer post decreased (Minsky and Turner 2015).

To compare the adhesion performance of our composite fibers to adhesives reported in literature, the adhesion stress of our DP composite mushroom fibers was calculated to be ca. 300 kPa. Recently, in a microscopic approach stiff mushroom fibers (diameter ca. 70–80, height 85–90µm) with a soft terminal layer of 4 and 7 µm thickness were studied and compared with the monolithic stiff fibers (Gorumlu and Aksak 2017). However, no significant difference on smooth surfaces was observed, since the adhesion decayed after the first measurement due to non-reversible fiber rupturing. Therefore, average adhesion values with ca. 77, 61, and 81 kPa were obtained for the monolithic, thin, and thick layer-coated mushroom patterns, respectively.

Finally, experiments were conducted to investigate the durability of our composite micropatterns. Both single printed (SP) and double printed composite fibers were subjected to cyclic adhesion experiments. The DP composite patterns exhibit a robust and high adhesion over more than 25 adhesion cycles, while the SP patterns show a decreased performance from 109 to 79 mN. Both composite patterns, SP and DP micropatterns, outperformed the film-terminated sample by two and seven times, respectively. Indeed, the adhesion of the DP patterns scattered slightly during the experiments. However, no gradual decrease could be observed and the adhesion performance recover within a few cycles. Microscopic observation showed that some fibers contacted each other upon strong stretching and quick releasing during the retraction of an adhesion cycle. However, the attachment was of temporary nature and fibers separated again within a few cycles and the adhesion performance was fully restored.

The gecko, our biological source of inspiration, showed a shear performance with two feet of ca. 90 kPa on a smooth surface (Irschick et al. 1996). Notably, the adhesion of the gecko foot-hairs is strongly coupled with an initial shear motion in order to reorient and align the adhesive tip-endings. Therefore, we compare our adhesives to the adhesion performance of the gecko under its optimal condition. Although our composite micropatterns surpass the performance of the gecko by three times on smooth surfaces, it is obvious that biological adhesive systems are developed for real world surfaces and enable animals to locomote over almost any surface, including rough surfaces. Initial experiments indicate that our composite micropatterns adhere to rough surfaces. Although the adhesion of DP composite fiber decreased from 217 to 39 mN, they outperform a flat S-PSA control sample with 28 mN on a rough surface (Fig. 3E and F). However, more experiments and detailed characterization need to be performed in the future.

In conclusion, we presented a novel approach for high-performance gecko-inspired adhesives. We demonstrated that microfibers with S-PSA mushroom tips outperform patterns with various tip geometries. The high adhesion strength of the composite microfibers was found to be due to the very thin S-PSA terminal tip layer and sharp tip edges, and thus improved load sharing. The optimal tip geometry and layer thickness, together with the covalent bonding enable high adhesion performance on smooth and rough surfaces with durability and repeatability, without any decay or irreversible deformation or fiber rupturing. Our concept of composite microfibrillar adhesives can provide significant benefits for a broad range of adhesion applications requiring high adhesion on various surfaces with different topographies. This includes wearable medical devices (Amjadi et al. 2016; Drotlef et al. 2017) enabling biocompatible and reversible adhesion to skin or other surfaces together with amplified signal transfer, transfer printing systems (Mengüç et al. 2012), and robotic manipulation (Song et al. 2017) capable of handling a wide range of complex and deformable objects.

## Materials and methods

### Fabrication of cylindrical microfibers decorated with different tip shapes

#### Cylindrical fibers

PDMS microfibers were obtained by replicating SU-8 lithographic templates as previously reported (del Campo et al. 2007). Sylgard 184 prepolymer and curing agent with weight ratio of 10:1 were mixed, degassed, and cast onto the SU-8 mold. The samples were cured in a vacuum oven at 90°C for 1 h and then demolded. Micropatterns with 45 µm tip diameter, 55 µm spacing, and 89 µm height were obtained.

#### Microfibers decorated with hemispherical S-PSA tips

Skin adhesive MG 7-9900 (Dow Corning) prepolymer and curing agent with weight ratio of 1:1 were mixed and degassed for 2 min. Next, a thin and homogeneous layer of S-PSA precursor solution with 25–30 µm thickness was coated over a glass plate by a film applicator (Multicator 411, Erichsen GmbH & Co. KG). After partial crosslinking (pre-crosslinking) of the S-PSA layer for 8 min, the micropatterned PDMS was manually inked onto the thin layer and placed upside down in a petri dish. The S-PSA was fully crosslinked after 12 h at room temperature and micropatterns with 45 µm tip diameter, 55 µm spacing, and 101 µm height with hemispherical tips were obtained.

#### S-PSA mushroom fibers

A thin and homogeneous layer of S-PSA precursor solution with 25–30 µm thickness was coated over a glass plate by a film applicator. After partial crosslinking of the S-PSA layer for 8 min, the micropatterned PDMS was manually inked onto the thin layer and placed on a perfluorinated silicon wafer. The S-PSA was fully crosslinked after 12 h at room temperature and peeled-off. Mushroom-shaped microfibers with 62 µm tip diameter, 31 µm spacing, and 92 µm height were obtained.

#### Film-terminated microfibers decorated with S-PSA

A thin and homogeneous layer of S-PSA precursor solution with 25–30 µm thickness was coated over a PET film by a film applicator. The micropatterned PDMS was manually inked onto the thin layer, crosslinked for 12 h at room temperature, and peeled-off. Film-terminated micropatterns with 99 µm height were obtained. Please note that the PET film is required in order to facilitate the detachment of the cured film-terminated patterns.

#### VS mushroom fibers

A thin and homogeneous layer of the VS precursor solution with 25–30 µm thickness was coated over a glass plate by a film applicator. After partial crosslinking of the VS layer for 30–45 s, the micropatterned PDMS was manually inked onto the thin layer and placed on a perfluorinated silicon wafer. Within a few minutes, the viscous VS was crosslinked, peeled-off, and mushroom-shaped microfibers with ca. 65 µm tip diameter, 35 µm spacing, and 93 µm height were obtained.

It should be noted that we employed VS for the fabrication of the mushroom fiber tips due to its fast crosslinking kinetics and facile processing, enabling PDMS microstructures with optimal and homogenous mushroom fiber tips. The slow crosslinking kinetics and high temperature curing of the PDMS may cause imperfect mushroom tips, leading to inhomogeneous patterns.

#### SP composite mushroom fibers

A thin and homogeneous layer of S-PSA precursor solution with 25–30 µm thickness was coated over a glass plate by a film applicator. After partial crosslinking of the S-PSA layer for ca. 15–20 min, the VS mushroom pattern was manually inked onto the thin layer and printed on a perfluorinated silicon wafer. The S-PSA was fully crosslinked after 12 h at room temperature and peeled-off. Composite mushroom-shaped microfibers with 70 µm tip diameter, 30 µm spacing, and 96 µm height were obtained.

#### Double printed composite mushroom fibers

VS mushroom patterns were manually inked onto the thin layer of S-PSA as described before, printed on a perfluorinated silicon wafer for 5 s, peeled-off, and printed on a pristine perfluorinated silicon wafer. The S-PSA was fully crosslinked after 12 h at room temperature and peeled-off. Please note that after the first printing and peeling process, S-PSA material was transferred from the fiber tips to the silicon wafer resulting in a reduced terminal layer thickness of the composite fibers. Composite mushroom-shaped microfibers with 67 µm tip diameter, 33 µm spacing, and 95 µm height were obtained.

#### Flat S-PSA control

A thin and homogeneous layer of S-PSA precursor solution with 500 µm thickness was coated over a glass plate by a film applicator and fully crosslinked for 12 h at room temperature.

### Experimental setup

A customized adhesion setup was built onto an inverted optical microscope (Axio Observer A1, Zeiss) with a video camera (Grasshopper^®^3, Point Grey Research Inc.) to visualize and record the contact interface. The adhesion force was recorded by a sensitive load cell (GSO-25, Transducer Techniques^®^) attached to a computer-controlled high-precision piezo motion stage (LPS-65 2″, Physik Instrumente GmbH & Co. KG) in *z*-direction. Fine positioning in *x*- and *y*-direction was done by a manual *xy*-stage (NFP-2462CC, Positionierungstechnik Dr Meierling) and tilt correction was adjusted by two goniometers (M-GON65-U, Newport). Motion control of the piezo stages and the data acquisition were achieved by a customized Linux code (Ubuntu™, Canonical Ltd). The program allowed to control preloads, velocities, displacements in *x*- and *z*-directions, and contacting time.

### Experimental procedure

Micropatterned samples were placed under a spherical glass probe with 4 mm radius. During the adhesion testing the indenter approached the sample surface at 50 µm/s and was first brought in contact with a preload of 50 mN. After a contact time of 10 s, the indenter was retracted at a speed of 50 µm/s until the probe was detached from the sample. The spherical indenter was cleaned after each measurement cycle with a particle-free tissue and isopropanol. The experiments were conducted in a temperature and humidity controlled lab and were in the range of 20–25°C and 25–35%, respectively. For each data point three samples with a minimum of five measurements were performed.

## Notes

From the symposium “The path less traveled: Reciprocal illumination of gecko adhesion by unifying material science, biomechanics, ecology, and evolution” presented at the annual meeting of the Society of Integrative and Comparative Biology, January 3–7, 2019 at Tampa, Florida.

## References

[icz009-B1] Aksak B, Sahin K, Sitti M. 2014 The optimal shape of elastomer mushroom-like fibers for high and robust adhesion. Beilstein J Nanotechnol 5:630–8. 24991499 10.3762/bjnano.5.74 PMC4077298

[icz009-B2] Amjadi M, Turan M, Clementson C, Sitti M. 2016 Parallel microcracks-based ultrasensitive and highly stretchable strain sensors. ACS Appl Mater Interfaces 8:5618–26. 26842553 10.1021/acsami.5b12588

[icz009-B3] Arzt E, Gorb S, Spolenak R. 2003 From micro to nano contacts in biological attachment devices. Proc Natl Acad Sci U S A 100:10603–6. 12960386 10.1073/pnas.1534701100 PMC196850

[icz009-B4] Autumn K, Liang YA, Hsieh ST, Zesch W, Chan WP, Kenny TW, Fearing R, Full RJ. 2000 Adhesive force of a single gecko foot-hair. Nature 405:681–5. 10864324 10.1038/35015073

[icz009-B5] Autumn K, Sitti M, Liang YA, Peattie A, Hansen W, Sponberg S, Kenny T, Fearing R, Israelachvili J, Full RJ. 2002 Evidence for van der Waals attachment for geckos. Proc Natl Acad Sci U S A 99:12252–6. 12198184 10.1073/pnas.192252799 PMC129431

[icz009-B6] Autumn K. 2006 In: Smith AM, Callow JA, editors. Properties, principles, and parameters of the gecko adhesive system. Biological adhesives. Berlin: Springer p. 225.

[icz009-B7] Carbone G, Pierro E. 2012 Sticky bio-inspired micropillars: finding the best shape. Small 8:1449–54. 22383385 10.1002/smll.201102021

[icz009-B8] Chung JY, Chaudhury MK 2005. Soft and Hard Adhesion. J Adhes 81:1119–45.

[icz009-B9] del Campo A, Greiner C, Álvarez I, Arzt E. 2007 Patterned surfaces with pillars with controlled 3D tip geometry mimicking bioattachment devices. Adv Mater 19:1973–7.

[icz009-B10] Del Campo A, Greiner C, Arzt E. 2007 Contact shape controls adhesion of bioinspired fibrillar surfaces. Langmuir 23:10235–43. 17722937 10.1021/la7010502

[icz009-B11] Drotlef DM, Amjadi M, Yunusa M, Sitti M. 2017 Bioinspired composite microfibers for skin adhesion and signal amplification of wearable sensors. Adv Mater 29:1701353–61. 10.1002/adma.201701353 28523760

[icz009-B12] Drotlef DM, Stepien L, Kappl M, Barnes J, Butt HJ, del Campo A. 2013 Insights into the adhesive mechanisms of tree frogs using artificial mimics. Adv Funct Mater 23:1137–46.

[icz009-B13] Drotlef DM, Blümler P, del Campo A. 2014 Magnetically actuated patterns for bioinspired reversible adhesion (dry and wet). Adv Mater 26:775–9. 24259374 10.1002/adma.201303087

[icz009-B14] Fischer SCL, Arzt E, Hensel R. 2017 Composite pillars with a tunable interface for adhesion to rough substrates. ACS Appl Mater Interfaces 9:1036–44. 27997118 10.1021/acsami.6b11642 PMC5235241

[icz009-B15] Fischer SCL, Kruttwig K, Bandmann V, Hensel R, Arzt E. 2017 Adhesion and cellular compatibility of silicone-based skin adhesives. Macromol Mater Eng 302:1600526–11.

[icz009-B16] Ge L, Sethi S, Ci L, Ajayan PM, Dhinojwala A. 2007 Carbon nanotube-based synthetic gecko tapes. Proc Natl Acad Sci U S A 104:10792–5. 17578915 10.1073/pnas.0703505104 PMC1904109

[icz009-B17] Gorumlu S, Aksak B. 2017 Sticking to rough surfaces using functionally graded bio-inspired microfibres. R Soc Open Sci 4:161105. 28680663 10.1098/rsos.161105 PMC5493905

[icz009-B18] Greiner C, del Campo A, Arzt E. 2007 Adhesion of bioinspired micropatterned surfaces: effects of pillar radius, aspect ratio, and preload. Langmuir 23:3495–502. 17315904 10.1021/la0633987

[icz009-B19] Irschick DJ, Austin CC, Petren K, Fisher RN, Losos JB, Ellers O. 1996 A comparative analysis of clinging ability among pad-bearing lizards. Biol J Linn Soc 59:21–35.

[icz009-B20] Kim S, Sitti M. 2006 Biologically inspired polymer microfibers with spatulate tips as repeatable fibrillar adhesives. Appl Phys Lett 89:261911–3.

[icz009-B22] Marvi H, Song S, Sitti M. 2015 Experimental investigation of optimal adhesion of mushroomlike elastomer microfibrillar adhesives. Langmuir 31:10119–24. 26322396 10.1021/acs.langmuir.5b02415

[icz009-B23] Mengüç Y, Yang SY, Kim S, Rogers JA, Sitti M. 2012 Gecko-inspired controllable adhesive structures applied to micromanipulation. Adv Funct Mater 22:1246–54.

[icz009-B24] Minsky HK, Turner KT. 2015 Achieving enhanced and tunable adhesion via composite posts. Appl Phys Lett 106:201604.

[icz009-B25] Murphy MP, Aksak B, Sitti M. 2009 Gecko‐inspired directional and controllable adhesion. Small 5:170–5. 19115348 10.1002/smll.200801161

[icz009-B26] Peisker H, Michels J, Gorb SN. 2013 Evidence for a material gradient in the adhesive tarsal setae of the ladybird beetle *Coccinella septempunctata*. Nat Commun 4:1661–7. 23552076 10.1038/ncomms2576

[icz009-B27] Prowse MS, Wilkinson M, Puthoff JB, Mayer G, Autumn K. 2011 Effects of humidity on the mechanical properties of gecko setae. Acta Biomater 7:733–8. 20920615 10.1016/j.actbio.2010.09.036

[icz009-B28] Puthoff JB, Prowse MS, Wilkinson M, Autumn K. 2010 Changes in materials properties explain the effects of humidity on gecko adhesion. J Exp Biol 213:3699–704. 20952618 10.1242/jeb.047654

[icz009-B29] Shahsavan H, Zhao B. 2014 Bioinspired functionally graded adhesive materials: synergetic interplay of top viscous-elastic layers with base micropillars. Macromolecules 47:353–64.

[icz009-B30] Song S, Drotlef DM, Majidi C, Sitti M. 2017 Controllable load sharing for soft adhesive interfaces on three-dimensional surfaces. Proc Natl Acad Sci U S A 114:E4344–53. 28507143 10.1073/pnas.1620344114 PMC5465890

[icz009-B31] Spolenak R, Gorb S, Gao HJ, Arzt E. 2005 Effects of contact shape on the scaling of biological attachments. Proc R Soc Lond Ser A 461:305–19.

